# Comparative Plastome Analysis of Root- and Stem-Feeding Parasites of Santalales Untangle the Footprints of Feeding Mode and Lifestyle Transitions

**DOI:** 10.1093/gbe/evz271

**Published:** 2019-12-17

**Authors:** Xiaoli Chen, Dongming Fang, Chenyu Wu, Bing Liu, Yang Liu, Sunil Kumar Sahu, Bo Song, Shuai Yang, Tuo Yang, Jinpu Wei, Xuebing Wang, Wen Zhang, Qiwu Xu, Huafeng Wang, Langxing Yuan, Xuezhu Liao, Lipeng Chen, Ziqiang Chen, Fu Yuan, Yue Chang, Lihua Lu, Huanming Yang, Jian Wang, Xun Xu, Xin Liu, Susann Wicke, Huan Liu

**Affiliations:** 1 BGI-Shenzhen, Shenzhen, China; 2 China National GeneBank, BGI-Shenzhen, Shenzhen, China; 3 State Key Laboratory of Agricultural Genomics, BGI-Shenzhen, Shenzhen, China; 4 State Key Laboratory of Systematic and Evolutionary Botany, Institute of Botany, Chinese Academy of Sciences, Beijing, China; 5 Fairylake Botanical Garden, Shenzhen & Chinese Academy of Sciences, Shenzhen, China; 6 School of Basic Medical, Qingdao University, China; 7 BGI-Qingdao, BGI-Shenzhen, Qingdao, China; 8 Hainan Key Laboratory for Sustainable Utilization of Tropical Bioresources, Institute of Tropical Agriculture and Forestry, Hainan University, Haikou, China; 9 College of Chinese Medicine Materials, Jilin Agricultural University, China; 10 MGI, BGI-Shenzhen, Shenzhen, China; 11 Institute for Evolution and Biodiversity, University of Muenster, Germany†These authors contributed equally to this work

**Keywords:** parasitic plants, Santalales, reductive plastome evolution, selection, feeding mode, Balanophoraceae

## Abstract

In plants, parasitism triggers the reductive evolution of plastid genomes (plastomes). To disentangle the molecular evolutionary associations between feeding on other plants below- or aboveground and general transitions from facultative to obligate parasitism, we analyzed 34 complete plastomes of autotrophic, root- and stem-feeding hemiparasitic, and holoparasitic Santalales. We observed inexplicable losses of housekeeping genes and tRNAs in hemiparasites and dramatic genomic reconfiguration in holoparasitic Balanophoraceae, whose plastomes have exceptionally low GC contents. Genomic changes are related primarily to the evolution of hemi- or holoparasitism, whereas the transition from a root- to a stem-feeding mode plays no major role. In contrast, the rate of molecular evolution accelerates in a stepwise manner from autotrophs to root- and then stem-feeding parasites. Already the ancestral transition to root-parasitism coincides with a relaxation of selection in plastomes. Another significant selectional shift in plastid genes occurs as stem-feeders evolve, suggesting that this derived form coincides with trophic specialization despite the retention of photosynthetic capacity. Parasitic Santalales fill a gap in our understanding of parasitism-associated plastome degeneration. We reveal that lifestyle-genome associations unfold interdependently over trophic specialization and feeding mode transitions, where holoparasitic Balanophoraceae provide a system for exploring the functional realms of plastomes.

## Introduction

Flowering plants contain many lineages that rely on organic carbon from either another plant or a fungal network (Wicke and Naumann 2018). The majority of such parasitic (heterotrophic) plants are still able to perform photosynthesis (PS). Some can even survive as free living organisms (facultative heterotrophs), whereas others are in need of a host plant (haustorial parasites) or fungal network (mycohetrotrophic plants) during at least certain lifestyle stages to fulfill their life cycle (obligate heterotrophs). Having completely lost the ability to photosynthesize, the holoparasitic lifestyle constitutes the end of the spectrum of obligate parasitic plants, where the parasite receives all nutrition and water from another plant. These lifestyle specializations are irrespective of the feeding type and occur both in stem- or root-feeding parasites, a classification that defines where the parasites attacks and infects their host (Westwood et al. 2010).

Relaxed constraints on plastid genes, especially in PS-related genes, lead to subtle or significant changes in plastomes of parasitic plants, including mycoheterotrophs, which both require essential organic carbon (reviewed in Wicke and Naumann 2018). The over 95 sequenced plastomes of parasites published to date (last access: May 2019) reveal convergent genome rearrangements and gene losses (Funk et al. 2007; McNeal et al. 2007; Barrett and Davis 2012; Wicke et al. 2013; Barrett et al. 2014; Petersen et al. 2015, 2018; Bellot et al. 2016; Logacheva et al. 2016; Naumann et al. 2016; Ravin et al. 2016; Samigullin et al. 2016), with some holoparasite plastomes being reduced to a mere 10% of the size of an average angiosperm plastid genome (Bellot and Renner 2016), or even possibly lost (Molina et al. 2014).

Studies of plastome evolution in heterotrophic plants of differing specializations provided a first understanding of the interdependent mechanisms of lifestyle and genomic transitions along the transition to a nonphotosynthetic lifestyle in plants. A conceptual model postulates that the transition to obligate parasitism relaxes selection constraints in a stepwise manner, whereby the evolutionary rates and selection pressure coevolve with macrostructural and microstructural changes, the extent of functional reduction and changes of lifestyle toward a new evolutionary equilibrium (Wicke et al. 2016; Wicke and Naumann 2018). This integrated concept provides testable hypotheses regarding the general interplay between large-scale genotypic and phenotypic changes along lifestyle transitions. Currently unaddressed are whether the root- and stem-feeding modes contributes to the series of functional and physical plastome reduction and to molecular evolutionary rate shifts.

The sandalwood order, Santalales, is one of the rare lineages, where autotrophic, hemiparasitic, and holoparasitic species occur within or (mostly) across closely related families (Nickrent et al. 2010; Su et al. 2015). Unlike other parasitic plant groups, Santalales also harbor both stem- and root-feeding parasites among its hemiparasitic members. Phylogenetic analyses have shown that stem-feeders, often referred to as the mistletoe habit, have evolved from root-parasitic ancestors five times independently, and brought about most of the generic and specific diversity seen in this order (Vidal-Russell and Nickrent 2008; Nickrent et al. 2010). Most of the parasites could be clearly classified as root- or stem-parasitic, but there are some species which have ambiguous parasitic mode, for example *Dendrotrophe varians* was recorded as root- or stem-parasites (Vidal-Russell and Nickrent 2008; Shin and Lee 2018), and *Tripodanthus acutifolius* form haustorial connections to both stems and roots of host (Vidal-Russell and Nickrent 2008). There are also some rare events of evolutionary reversions or atavism in which the stem-parasites evolved back into root-parasites (e.g., *Nuytsia*, *Atkinsonia*, and *Gaiadendron* in Loranthaceae). The marked evolutionary bias from root- to stem-feeding forms indicates that stem-parasitism provided an enormous eco-evolutionary advantage, potentially leaving footprints of positive selections in the parasite’s genomes (Vidal-Russell and Nickrent 2008).

Circumstantial evidence suggests that the transition from root- to stem-feeding forms coincides with an increasing parasitic (trophic) specialization. Some root-parasites were reported to survive even without a host (e.g., in Santalaceae; Veenendaal et al. 1996; Kuijt and Hansen 2015), whereas stem-parasites might germinate in the absence of a host but then completely rely on a host to complete their life cycle (Kuijt and Hansen 2015). Severe and convergent morphological modifications such as the reduction of leaves to scale-like structures in some parasitic Misodendraceae and Viscaceae indicate that trophic specialization increases over time (Vidal-Russell and Nickrent 2008; Maul et al. 2019). An increasing dependence on the host has also been based on the reduction of chlorophyll content (Hull and Leonard 1964b; De La Harpe et al. 1979, 1981). Comparisons of heterotrophic carbon acquisition between root- and stem-parasites revealed a relatively high heterotrophic carbon acquisition in stem-feeders (Hull and Leonard 1964a; Těšitel et al. 2010; Bell and Adams 2011; Kuijt and Hansen 2015). Hence, we can hypothesize that ancestral root-parasitism in Santalales shows fewer symptoms of the plastid genomic parasitic reduction syndrome than stem-feeders. A systematic comparison of root- and stem-parasites might provide a proxy to uncover the elusive patterns underlying the subtle transition between mild and intensified parasitism.

Previous analysis in plastomes of hemiparasitic plants in Santalales also indicated the different levels of genome size reduction and gene loss (Petersen et al. 2015; Li et al. 2017; Shin and Lee 2018). The selection pattern of remaining genes was different compared with other dicots, but no significant difference was found within Santalales plastomes (Petersen et al. 2015). Correlation between gene content and type of parasitism (obligate/facultative and stem-/root-parasites) was not found either (Shin and Lee 2018).The insignificance or uncorrelation may be due to the limited samples analyzed. The holoparasitic plants in Balanophoraceae showed miniaturized plastomes, extremely reduced gene contents, high AT contents, and novel genetic codes, uncovered the extreme plastome evolution in this fast-evolving lineage (Su et al. 2019; Schelkunov et al. 2019).

We here present an evolutionary analyses of 34 autotrophic, hemiparasitic, and holoparasitic Santalales plastomes to unravel the subtle changes from root- to stem-feeding parasites. To disentangle interdependencies between genetic and lifestyle traits, we employed a battery of phylogenomic comparative methods in combination with trait-rate fusion models, allowing us to show that the reduction of plastid genes occurs alongside nucleotide substitution rate elevation and changes in selection pressure. In contrast, the extreme reconfigurations we observe in holoparasitic Santalales have apparently undergone a different evolutionary trajectory. Our findings provided a more detailed view of the molecular evolutionary mechanisms along the transition from autotrophy to parasitism in plants and, for the first time, enlighten the contributions of different parasitic modes on reductive plastome evolution.

## Materials and Methods

### Plant Sampling, DNA Extraction, and Sequencing

In addition to the 17 Santalales species with publicly available plastid genomes (Petersen et al. 2015; Li et al. 2017; Shin and Lee 2018; Su et al. 2019), we newly sequenced 17 hemiparasitic species (4 root-parasites and 13 stem-parasites) and 2 holoparasites belonging to the sandalwood order (supplementary table S1, Supplementary Material online). Total genomic DNA was extracted from leaves or inflorescences using a CTAB-based DNA extraction protocol. High-quality DNA extracts were then used to construct 150–250 bp insert sizes libraries with MGIEasy DNA Library Preparation Kit according to the manufacturer’s instructions and sequenced on a BGISEQ-500 sequencer at the BGI Shenzhen in paired-end mode with read lengths of 100 bp, each producing 60∼100 G clean data. DNA of *Balanophora fungosa* subsp. *indica* was used to construct an SMRT sequencing library with an insert size of 10 kb. This library was sequenced using the PacBio Sequel system (Pacific Biosciences, Menlo Park, CA) at BGI, Wuhan. In addition, whole-genome shotgun data of *Santalum album* was retrieved from NCBI (SRA: SRR5150443) and analyzed together with all other samples.

### Assembly and Annotation

For those samples with BGISEQ-500 data, we assembled plastomes using the de novo assembler NOVOPlasty v2.7.0 (Dierckxsens et al. 2017) with a k-mer size of 31. Two different genes were used as seeds from which we reconstructed plastid genome sequence by iterative extension, the large subunit of RuBisCO (*rbc*L) from *Taxillus chinensis* (KY996492) was used for 17 hemiparasites, and acetyl-CoA carboxylase subunit D gene (*accD*) from *Balanophora reflexa* (KX784266) was used for 2 holoparasitic *Balanophora* species. We assembled the plastome of *Balanophora fungosa* subsp. *indica* with CANU v1.8 (Koren et al. 2017) using the pacbio data, and 50 G WGS data from BGISEQ-500 were used for correction.

To assess the coverage and completeness of the assemblies, we mapped ∼3 Gb of randomly selected paired-end reads for each species to the corresponding assembly. This strategy allowed us to define the molecule type and locate the large inverted repeat (IR) boundaries properly in our reference taxon (supplementary fig. S1, Supplementary Material online). Meanwhile, a two-way comparison of six published plastomes with our own identified as different accessions of the same species was constructed with BlastN v 2.2.25 (http://blast.ncbi.nlm.nih.gov) to assess the assembly results. The two assembly versions of *Balanophora fungosa* subsp. *indica* were also compared.

Coding regions were extracted from the completed annotations with genBlastA v1.0.1 (She et al. 2008) and genewise v2.4.1 (Birney et al. 2004) using a custom plastome data set containing 28 reference species (supplementary table S2, Supplementary Material online). The start and stop codons, and short exons were manually identified through BlastN. Based on function, we divided the coding regions into three categories: housekeeping (HK), photosynthesis (PS), and other genes. The accurate in silico determination of start and stop codons as well as the resolution of exon–intron boundaries were assisted with transcriptome data of six parasitic Santalales species (supplementary table S2, Supplementary Material online) analyzed by the 1KP project (https://db.cngb.org/onekp/).We classiﬁed genes as pseudogenes when they had frameshifts and/or internal stop codons. Ribosomal RNA genes were identified by BlastN v 2.2.25 (http://blast.ncbi.nlm.nih.gov) searches against our custom plastome database (see above, supplementary table S2, Supplementary Material online). Finally, tRNAscan-SE v1.21 (Lowe and Eddy 1997) was used to further verify tRNA genes.

### Phylogenetic Analysis

To reconstruct phylogenetic relationships of our study group, we concatenated our single-gene alignments to one super alignment of 61 genes present in 25 out of 36 species when Balanophoraceae was included. Another alignment with 42 genes which were present in 31 species excluding Balanophoraceae was used for phylogeny analysis. This data matrix was subjected to tree building with maximum likelihood and Bayesian inference using the programs PhyML v3.0 (Guindon et al. 2010) with 1,000 bootstrap replicates, and MrBayes v3.1.2 (Ronquist and Huelsenbeck 2003) with 100,000 generations.

### Comparative Plastid Genome Analysis

Pairwise whole-genome alignments were generated by LASTZ v 1.02.00 (Harris 2007) with the following settings: T = 2, C = 2, H = 2,000, Y = 3,400, L = 6,000, K = 2,200. The main chains of the two selected genomes were calculated by chainNet (https://bioconda.github.io/recipes/ucsc-chainnet/README.html), which links aligned segments into larger structures. Based on these pairwise segments, we built a multiple-way alignment using MULTIZ v1.0 (Blanchette 2004) as a basis for further comparative analysis like the identification of collinear blocks, indel occurrences, or to compute evolutionary distances.

Repetitive elements were investigated using REPuter (Kurtz 2001), which we ran with a Hamming distance equal to three, an e-value of 10e-3, and 20 bp of minimum repeat size. Before statistical analysis, overlapping repeats were merged into one, where possible.

### Molecular Evolutionary Analyses of Plastid Genes

We employed the program CodonW v1.4.4 (http://codonw.sourceforge.net/) for the analyses of codon usage and nucleotide composition in plastid coding regions. To evaluate differences in A, T, C, and G distribution, total GC content, and GC content at different codon positions, we performed pairwise Wilcoxon tests with sequential alpha-error correction in *R* v3.2.1.

To infer how pseudogenes or fragmented genes degraded, we examined the coding regions (CDS) from 34 plastid genomes individually, based on single-gene alignments performed with Clustal X 2.0 (Larkin et al. 2007). The variable and unambiguous positions per gene region were traced over the phylogeny in MACCLADE v3.51, in addition to tracing the number of nucleotide substitutions and simple-coded indels, which were encoded with SEQSTATE v.1.4.1 (Müller 2005), over the phylogenetic tree.

Nonsynonymous (d*N*) and synonymous substitution rates (d*S*) and selection intensity (*k*) in retained plastid genes were analyzed using HYPHY v.2.2 (Kosakovsky Pond et al. 2005), and tests were run by gene and by functional gene class. Relative d*N* and d*S* were analyzed by likelihood ratio tests under the MG94-GTR hybrid model with a 3×4 codon frequency matrix.

To analyze interspecies changes of selectional strength, we used a branch-site random effects likelihood method with RELAX (Wertheim et al. 2015) as described in Wicke et al. (2016). Different test branch sets were evaluated by using Akaike weights to identify the best lifestyle modes for each gene or gene sets. Based on a branch-site random effects likelihood method to test for selection intensity, the RELAX framework uses a parameter, *k*, to test whether and how *ω* deviates from neutrality (i.e., *ω*  =  1). As relaxation of selection distinctly affects sites under purifying selection (*ω*  <  1) and sites under positive selection (*ω*  >  1), it will move *ω* toward 1 if selection is relaxed (i.e., *ω* < 1 increases and *ω* > 1 decreases). Using partitioned reference and test branches in a given tree, the null model assumes *k* = 1 for all branches (i.e., test and reference branches have the same *ω* distribution), whereas in the alternative model, *k* is allowed to differ for the reference and test branch set.

We tested for correlations between pairs of continuous genomic traits (genome size, GC content, repeats density, gene number, and indels density in genes) and discrete trait (lifestyle and feeding mode) using phylogenetic ANOVA implemented as function “phylANOVA” in the R package “phytools” (Revell 2012). phylANOVA is based on phylogenetic simulation. It takes into account species relationships by considering the phylogeny as a covariate when analyzing the associations of a dependent factor, here lifestyle and feeding mode, with various continuous traits (genome size, GC content, repeats density, gene number, and indels density in genes).

Coevolution of genomic traits (as above) with various parameters of the nucleotide substitution process (d*S*, d*N*, *ω*), and lifestyle or the feeding mode were analyzed with COEVOL v.1.4 (Lartillot and Poujol 2011) as described in Cusimano and Wicke (2016).

## Results

### Plastid Genome Structure

**Fig. 1.**
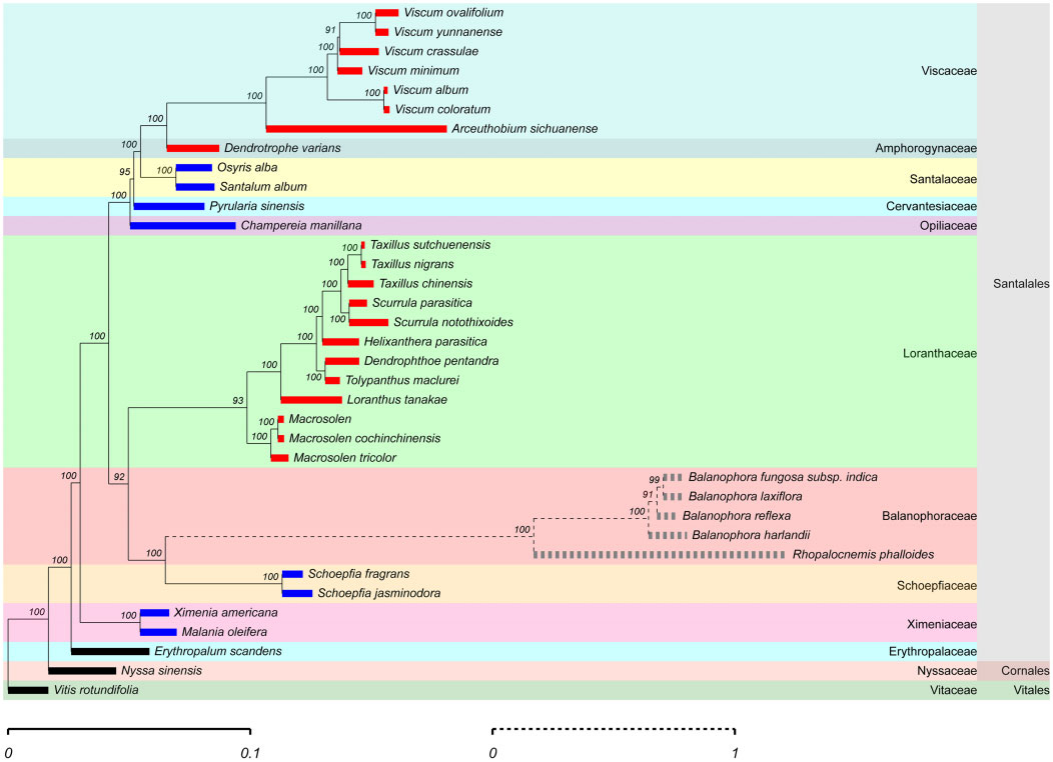
—Phylogeny of nonparasitic and parasitic Santalales. The phylogenetic tree has been inferred with Bayesian methods and a concatenated data set of seven plastid genes shared between all study taxa. The scale of the phylogram is in substitutions per site. The branch colors indicate lifestyle and parasitic specialization (black: autotrophy; blue: root-feeding hemiparasites; red: stem-feeding hemiparasites; gray: holoparasites). Note.—In this article, *Dendrotrophe varians* is classified as stem-feeding parasites as where the plastome data come from Shin and Lee (2018).

Genome skimming along with the seed-and-extend algorithms was the basis for us to complete plastid genomes for 19 parasitic plants from the Santalales to complement the existing data of 15 taxa from ten families (fig. 1 andsupplementary table S1, Supplementary Material online). A comparison of six published plastomes with our own revealed a general high identity between different accessions of the same species (supplementary fig. S2, Supplementary Material online). For example, the 2 plastomes of *Taxillus chinensis* are 99.93% identical, and they differ only by 5 mismatches and 17 gaps over 75 bases. Taken together, these results indicate that our bioinformatic approach was suitable to generate plastid genome assemblies of high quality.

Plastid genome size in our Santalales study taxa ranged between 15.5 and 156.8 kb in length (supplementary table S3, Supplementary Material online). Photosynthetic (chlorophyllous) Santalales all exhibited a normal quadripartite architecture and were mostly collinear with plastomes of autotrophic plants (supplementary fig. S3*a*, Supplementary Material online), whereas the holoparasitic Balanophoraceae showed substantial plastid genomic reconfigurations (supplementary figs. S3*b* and S4, Supplementary Material online). The set of intact and commonly present genes (explained in detail below) enabled reconstructing the phylogenetic relationships among our study taxa (fig. 1). Both Bayesian inference and maximum likelihood analyses yielded well-resolved trees with high congruency between the different methods, which provided the basis for downstream genomic analysis in phylogenetic context.

The plastome sizes of parasites were smaller than those of autotrophs, with the exception of the root-parasitic *Ximenia americana*, whose plastome of 156.8 kb length was larger than those of the analyzed autotrophs due to expanded IRs; we found the smallest plastome (107.5 kb) of investigated hemiparasitic Santalales in *Arceuthobium sichuanense* (Viscaceae sensu Nickrent et al. 2010; Su et al. 2015). The plastomes of Balanophoraceae are compact with overlapping genes, highly reduced spacers, and shrunken protein genes. Although the four *Balanophora* species show similar in plastid genomic architecture, *Rhopalocnemis phalloides* differs in its coding capacity and gene order (supplementary fig. S4, Supplementary Material online). The genomic reductions are significantly associated with lifestyle transitions, for example, from free-living plants to both hemiparasitism and holoparasitism (phylAnova and posthoc *P* values: < 0.05; table 1). We observed several notable deletions or insertions in, mostly, the IR regions, leading to reductions, extensions, or translocation of the boundaries between the IRs and the small single copy (SSC) region. For example, the fragment normally constituting the SSC, which in *Vitis rotundifolia* is part of the IR region, was completely lost in *Pyrularia sinensis* (Santalaceae) and a commonly IR-located fragment became the SSC. In the large single copy region, we identified the ∼24 kb inversion known from *Viscum minimum* (Petersen et al. 2015) also in *Viscum yunnanens* (supplementary fig. S3, Supplementary Material online).

**Table 1:** Results of Genomic Trait-Lifestyle Variance Analysis in Phylogenetic Context

		posthoc					
Type	phylAnova	NP-HP	NP-RH	NP-SH	RH-HP	SH-HP	RH-SH
Genome size	**0.016**	**0.036**	**0.006**	**0.015**	**0.048**	**0.048**	0.1
GC content	**0.001**	**0.006**	0.913	0.254	**0.006**	**0.006**	0.201
Gene no.	**0.011**	**0.02**	**0.006**	**0.006**	**0.036**	**0.036**	0.571
Repeat density	0.515	—	—	—	—	—	—
Indel density	0.189	—	—	—	—	—	—

NP, nonparasite; SH, stem hemiparasite; RH, root hemiparasite; HP, holoparasite. Significant values are in bold.

Our analyses also revealed that all photosynthetic species have a GC content of 34.9–38.2%, and is although unevenly distributed across the plastome. With between 22.6% in *Viscum yunnanens* and 46.3% in *Pyrularia sinensis*, GC contents varied mostly in the SSC regions of stem-feeding parasites, in part relating to the IR/SSC shifts (supplementary table S4, Supplementary Material online). In Balanophoraceae, the GC content drops to between 11.6% and 13.2%. This extraordinary nucleotide compositional bias is notably related to the evolution of parasitism (phylAnova *P* value = 0.001), whereby posthoc tests indicate that only holoparasitism is a main factor rather than the emergence of hemiparasitism or feeding-mode transitions (table 1).

### Functional Reduction

Plastomes of photosynthetic Santalales encode between 77 and 101 genes (supplementary table S4 and fig. S5, Supplementary Material online), of which 52–70 are protein-coding genes, 21–30 tRNAs, and 4 rRNAs. We observed no physical loss of plastid genes in *Erythropalum scandens* compared with non-Santalalean autotrophs. All of the 28 analyzed hemiparasites have lost the plastid NADH dehydrogenase complex (*ndh* genes), most were absent from their plastomes (supplementary data set S2, Supplementary Material online). The plastid translation initiation factor 1 (*infA*) gene was only intact in early divergent root-hemiparasites *Malania oleifera*, *Ximenia americana*, and our two *Schoepfia* species (Olacaceae), but was lost in all other root- and stem-feeding hemiparasites. The ribosomal protein genes *rpl32*, *rps15* were lost in *Malania oleifera*, *Pyrularia sinensis*, *Arceuthobium sichuanense*, and all Loranthaceae, the latter also all lacking a plastid *rps16* gene and, except for the *Macrosolen* species and *Dendrophthoe pentandra* that retain a pseudogenized copy of *rpl16*. None of the examined Viscaceae species retained the ribosomal protein gene *rpl33*, and one species *(Arceuthobium sichuanense*) also lacked all four genes for the plastid-encoded polymerase (PEP). Several genes (e.g., *ycf1*, *ccsA*, and *psaC*) were randomly deleted from the plastomes of a few species. Most notably, several tRNAs were lost in most hemiparasites, and, according to our DNA sequence data, the *clpP* gene, which encodes a proteolytic subunit of an ATP-dependent protease, appears to be a pseudogene in *Santalum album* (Santalaceae), *Pyrularia sinensis*, *Arceuthobium sichuanense*, and all Loranthaceae, except for *Taxillus sutchuenensis*. In sum, these results are in line with earlier reports of the progression of the parasitism-related plastome reduction while they also uncover new, perhaps Santalales-specific losses of some plastid HK genes.

Compared with hemiparasites in Santalales, plastomes of holoparasitic Balanophoraceae showed the most dramatic functional reductions as previously reported (Su et al. 2019; Schelkunov et al. 2019), with only 14–18 HK genes annotated, no photosynthetic genes or tRNA genes for protein synthesis, and 3 rRNA genes in the four *Balanophora* species, while only 1 rRNA gene in *R. phalloides*. As previously reported in two *Balanophora* species (Su et al. 2019), the loss of introns were also found in the two newly assembled *Balanophora* species, for example, two *clpP* introns, *rpl2* intron, while the *trans*-spliced intron 1 of *rps12* was retained but intron 2 lost (supplementary fig. S4, Supplementary Material online). Interestingly, all these introns were present in *R. phalloides* (supplementary fig. S4, Supplementary Material online), which indicated that the intron loss events happened after divergence of the ancestors of *Balanophora* and *Rhopalocnemis*. Functional reduction coincides primarily with the evolution of hemiparasitism and holoparasitism in Santalales (phylAnova and posthoc test *P* values < 0.01; table 1). No significant difference in gene number variance exists between root- and stem-feeding hemiparasites (phylAnova, posthoc test *P* value: 0.571; table 1).

### Nucleotide Compositional Bias and Codon Usage

Compared with autotrophs like *Vitis rotundifolia* (Vitaceae), *Nyssa sinensis* (Nyssaceae), and *E. scandens*, the stem-feeding hemiparasites of Santalales showed up to 3.1% lower GC content, especially in Viscaceae (supplementary table S3, Supplementary Material online). We observed no nucleotide compositional difference in root-feeding hemiparasites. Our analysis of the minimal though notable drop of GC content in coding regions of stem-feeders (supplementary fig. S6 and table S4, Supplementary Material online) revealed differences in the GC-related usage of nucleotides between Loranthaceae and Viscaceae, whereby the former used more GC bases and the latter used less GC bases at the third codon than first and second codon sites (supplementary fig. S6, Supplementary Material online).

Comparing the nucleotide frequencies of codons between autotrophs, root- or stem-feeding hemiparasites, and holoparasites in phylogenetic context revealed that 29 positions in 11 genes show significant changes between autotrophs and root-feeding parasites (supplementary data set S2-1, Supplementary Material online), between autotrophs and stem-feeding hemiparasites (supplementary data set S2-2, Supplementary Material online), 68 positions in 22 genes are affected. And meanwhile, 53 positions in 18 genes differ between root- and stem-feeding hemiparasites (supplementary data set S2-3, Supplementary Material online). Most of the significant nucleotide composition change in photosynthetic genes. Balanophoroaceae, which all are holoparasites, differ from autotrophs and hemiparasites in 67 positions in all their retained genes (supplementary data set S2-4, Supplementary Material online). Together, our results indicate that more changes occur in the nucleotide composition when transferred from root-feeding to stem-feeding hemiparasites in photosynthetic genes. The retained HK genes in holoparasites show a notably different nucleotide usage that is concordant with their extremely high AT contents.

### Repetitive Plastid DNA

We analyzed the occurrence of different types of repeats and found that, compared with autotrophic non-Santalales species, *E.scandens* had a higher density of repeats (one repeat every 1.08 kb vs. one in ∼1.5 kb in non-Santalales references). Repeat density was slightly lower in root-feeding hemiparasites (lowest in *Schoepfia jasminodora* with one repeat per 3.04 kb), although that of *Ximenia americana* (one repeat per 0.48 kb) was in the same range as stem-parasites (one repeat per 0.53–1.22 kb in Loranthaceae and one repeat per 0.25–0.66 kb in Viscaceae) (supplementary table S5, Supplementary Material online). An analysis of variance in phylogenetic context found no significant differences in repeat densities between autotrophs, hemiparasites of different feeding forms, and holoparasites in Santalales (phylAnova *P* value = 0.515; table 1). Similarly, also variation in the number of small insertion and deletions (indels) is not primarily linked to transitions of lifestyle or feeding modes (phylAnova *P* value = 0.189; table 1). Forward (direct) and palindromic (inverted) repeats of mostly 20–30 nt lengths were the most dominant type in species with low amounts of repetitive plastid DNA. Mild differences in repeat densities were mostly due to an accumulation of reversed and complement repeat motifs and those longer than 31 bp (supplementary figs. S7 and S8, Supplementary Material online). Self–self alignments (supplementary fig. S9, Supplementary Material online) revealed that the higher repeat density in stem-parasites compared with nonparasites and root-parasites often, but not exclusively, accumulate around the IR-SSC junctions and near the center of the large single copy region. In contrast, repeats were homogenously dispersed in the autotrophic plants.

### Nucleotide Substitution Rates

**Fig. 2.**
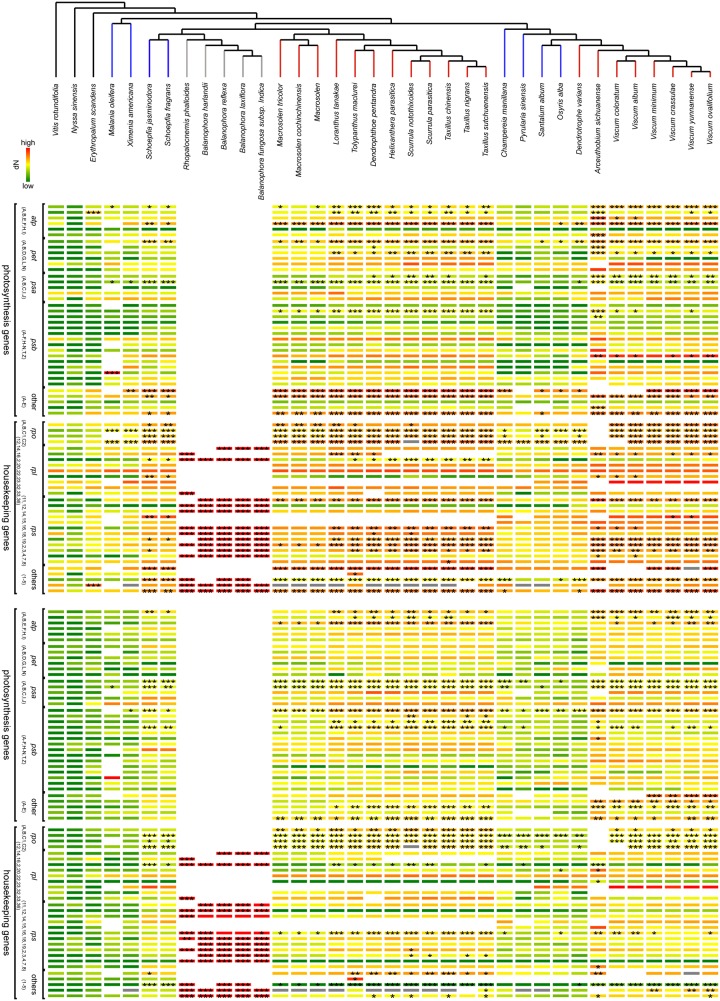
—Nucleotide substitution rate variation in plastomes of Santalales. Heatmaps illustrate the differences in log-transformed (*a*) nonsynonymous (d*N*) and (*b*) synonymous (d*S*) rates for each plastid protein-coding gene, whose names are provided from top to bottom per gene class. A phylogenetic tree on top indicates their relationships, and the colors of branches also show lifestyle and parasitic specialization the same as figure 1. Low rates are shown in green and high rates in red. Asterisks indicate the significance of rate difference as evaluated by Likelihood ration tests against the nonparasitic reference *Vitis rotundifolia* (*P < 0.05, **P < 0.01, ***P < 0.001). Abbreviations: other PS, other photosynthesis genes (A, *ccsA*; B, *cemA*; C, *rbcL*; D, *ycf3*; E, *ycf4*); other HK, housekeeping and metabolic genes (1, *matK*; 2, *infA*; 3, *ycf2*; 4, *clpP*; 5, *accD*).

Gene-by-gene analysis of nucleotide substitution rates showed no significant differences between the root-hemiparasitic Olacaceae and the nonparasitic *E.scandens* (fig. 2*a* and *b*). The genes *atpA*, *psaB*, *cemA*, *rpoA*, and *matK* evolve at elevated substitution rates in root-parasitic *Schoepfia* as well as in the analyzed species from Opiliaceae, Cervantesiaceae, Santalaceae, and Amphorogynaceae. An elevation of nucleotide substitution rates in the majority of PS and HK genes of stem-parasitic Loranthaceae was mainly restricted to the tribe Lorantheae. In contrast, high rates of nonsynonymous and synonymous substitutions across all functional gene classes generally characterize the stem-feeders of Viscaceae. The most extremes rates of molecular evolution occur in all plastid genes of Balanophoraceae, thus reflecting their extreme plastid genome structure (see above).

### Changes in Selection

**Fig. 3.**
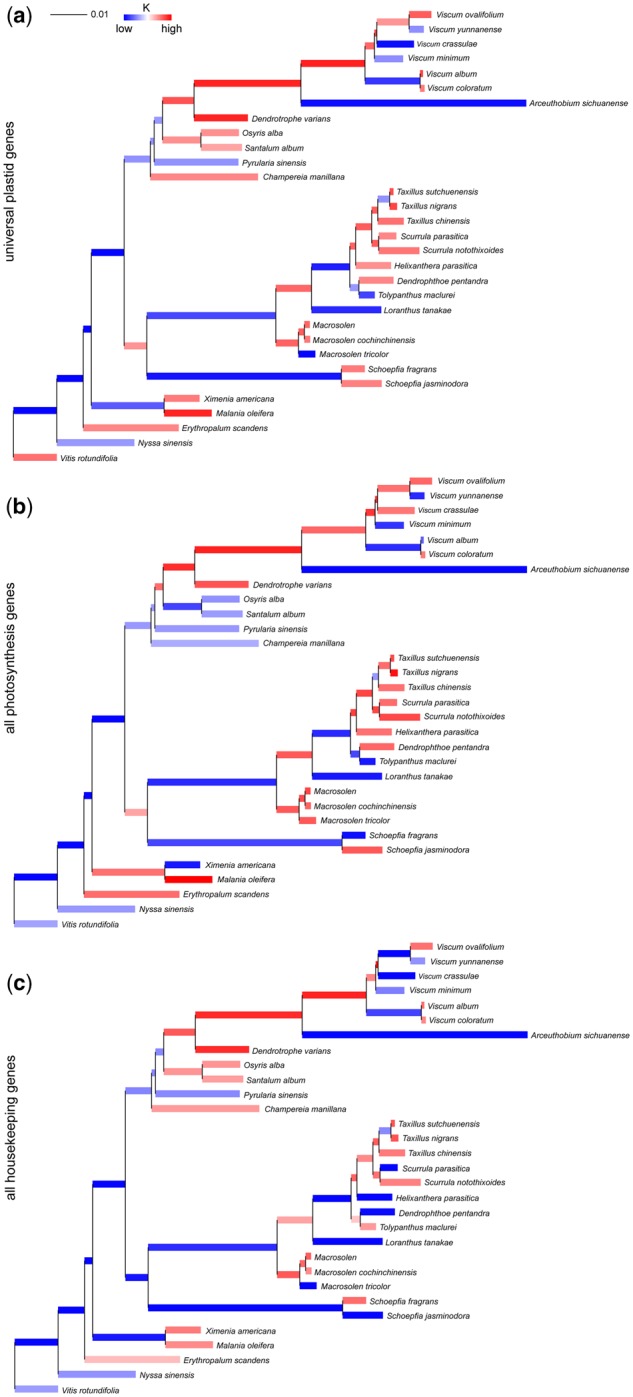
—Changes of selectional regimes excluding Balanophoraceae. Trees with colored branches highlight selectional changes per branch across (*a*) all universal genes, (*b*) photosynthetic genes, and (*c*) all housekeeping genes. The genes were color-coded according to the selection strength parameter *k*, inferred under the general descriptive *RELAX* model. Low *k* (blue) indicates a relaxation of purifying selection, whereas high *k* (red) suggests selection intensification. About 42 genes shared in these samples were used for construction of phylogenetic tree.

Selectional strength has been relaxed around the divergence of root-parasitic from autotrophic Santalales, in the last common ancestor of Schoepfiaceae and Loranthaceae, and the last ancestor of Opiliaceae, Cervantesiaceae, Santalaceae, Amphorogynaceae, and Viscaceae. We observed hardly any significant selectional shift in terminal lineages, that is, evolutionarily more recently, although some genes of *Viscum crassulae*, *Arceuthobium sichuanense*, *Macrosolen tricolor*, and *Loranthus tanakae* (Loranthaceae) all showed footprints of selection relaxations and *Dendrotrophe Varians* and *Malania Oleifera* experienced an intensification of selection (fig. 3*a and*supplementary data set S3, Supplementary Material online).

In Schoepfiaceae and Loranthaceae, PS genes have evolved under intensified selection after an ancestral relaxation of purifying selection along the transition from root- to stem-parasitism. In Opiliaceae, Cervantesiaceae, Santalaceae, Amphorogynaceae, and Viscaceae, there were no significant selectional shifts in extant lineages of root-parasitic species, while in stem-parasitic Viscaceae, the selection intensity was intense at ancestral (deeper) nodes, whereas extant lineages show relaxed selective constraints in PS genes (fig. 3*b*). In HK genes, it showed relaxed selective constraints at ancestral nodes, while there was no significant selectional shift in terminal lineages of root-feeding hemiparasites, but showed relaxation in some stem-feeding hemiparasites (fig. 3*c*).

We inferred the relative contribution of each major selectional shift to transitions in lifestyle and parasitic specialization and found that genes for PS experienced a significant shift of selection in stem-feeding parasites compared with nonparasites and root-feeding hemiparasites. With regards to the HK genes, we observed relaxations of selection in most genes when transitioning from the root- to stem-feeding mode (supplementary data set S3, Supplementary Material online). These results indicate that the transition from root- to stem-parasitism altered the overall selectional regime of plastid genes in Santalales, although no further genomic differences between the two feeding modes exists.

The studied species in Balanophoraceae all evolve under high levels of purifying selection (d*N*/d*S* = *ω* = 0.21–0.66). Most genes encoded Ribosomal proteins showed relaxed selection in the common ancestor of Balanophoraceae, including *rpl2*, *rps7*, *rps12*, *rps14*, *rps18*, while *accD* showed intensified selection (supplementary fig. S10, Supplementary Material online). Analyzing the contribution of lifestyle shifts (i.e., nonparasitism to parasitism to hemiparasitism to holoparasitism) to changes of selection revealed a significant shift from the hemiparasites to holoparasites (supplementary data set S4, Supplementary Material online).

### Coevolution of Molecular Evolutionary Rates, Genetic Traits, and Lifestyle

We analyzed the coevolution between shifts in evolutionary rates (d*N*, d*S*) and selection (*ω*), changes of genetic traits (genome size, GC content, repeat, gene content, indels), the parasitic specialization (lifestyle), and feeding mode (table 2). Among genetic traits, we observed the strongest association between high GC contents and low repeat densities, more genes, and fewer indels, all of which cocorrelating with parasitic specialization. Expectedly, our analyses also showed that larger plastomes tend to have high GC content (ppMC = 1, ppMCC = 1) and more repeats (ppMC = 1, ppMCC = 1). The strong correlations between the various phenotypic and genotypic factors suggest that the lifestyle and feeding mode transitions left marked footprints in nucleotide composition and plastome reduction.

**Table 2:** Posterior Probabilities (pp) from the Analysis of Genetic Coevolution

Trait	d*S*	d*N*	*ω*	Size^a^	GC	Repeat	Gene	Indel	Ls&FM^b^
	Marginal correlation (ppMC)								
d*S*	—	1.00	0.0021	1.00	1.00	1.00	0.9600	1.00	1.00
d*N*	0.96	—	NA	1.00	1.00	1.00	0.9600	1.00	1.00
*Ω*	0.031	NA	—	0.0001	0.0003	0.68	0.0003	0.069	0.013
Size^a^	0.72	0.33	0.66	—	1.00	1.00	1.00	1.00	1.00
GC	0.43	0.68	0.25	1.00	—	1.00	1.00	1.00	1.00
Repeat	0.13	0.79	0.70	1.00	0.038	—	0.99	1.00	1.00
Gene	0.31	0.25	0.27	0.46	1.00	0.70	—	0.99	0.99
Indel	0.97	0.22	0.34	0.82	0.12	0.68	0.69	—	1.00
Ls&FM^b^	0.25	0.80	0.54	0.21	1.00	0.99	0.0089	1.00	—
	**Maximally controlled correlation (ppMCC)**								

Note.—pp toward 1 indicates a positive correlation; pp toward 0 indicates a negative correlation.

^a^ Genome size.

^b^ Lifestyle and feeding mode, the parasitic lifestyle transition means that plants procure some or all of their nutrients from other living plants (i.e., nonparasitism to facultative parasitism to obligate parasitism to holoparasitism), and the feeding modes represent root- or stem-feeding forms in Santalales.

## Discussion

Being one of the largest groups of parasitic plants, Santalales harbor the most diverse set of hemiparasites whose lifestyle transitions and specializations on different feeding modes (root- vs. stem-feeders) offer the unique chance to uncover the eco-evolutionary contributions on early stages of lifestyle-induced functional and physical plastome reduction. By examining various root- and stem-feeding parasites, our study fills a gap in that it provides complete plastid genome data of hemiparasitic plants with different feeding modes, which are still underrepresented in the study of reductive plastome evolution. We examined plastid genome reduction over feeding mode and lifestyle transitions in Santalales based on a phylogenetic framework of 34 species that differed only from a previously established relationships within the sandalwood order (Nickrent et al. 2010; Su et al. 2015) by the position of Balanophoraceae to Schoepfiaceae (fig. 1).

Our results show that Santalales parasites of a mistletoe-habit are not significantly different in their plastid genome structure or size with root-feeding hemiparasites, although shifts of selectional constraints in plastid genes exist between both groups. It is well known that stem-feeding Santalales parasites are unable to survive without a host, have low net PS rates (Popp and Richter 1998) and have experienced independent reductions of the photosynthetic surface (Maul et al. 2019). Therefore, by considering this fact, we can conclude that functional plastome evolution in Santalales mirrors a trophic transition from facultative to obligate parasitism that accompanies the feeding-type transitions in this order, however, large-scale genomic reconfigurations in stem-feeding Santalales parasites is still lagging behind. Above that, our comparative analysis of plastid genomes in Santalales uncovered novel evolutionary trajectories that highlight the strong lineage-specific manner of gene losses, further enhancing our understanding of the erratic nature of functional reduction of genetic elements associated with HK functions.

Generally, heterotrophic plants, haustorial parasites, and mycoheterotrophs alike show a strong correlation between functional and physical reductions (Wicke and Naumann 2018). Reductive plastome evolution in parasites is strongly associated with lifestyle transitions and interdependent changes of molecular evolutionary rates, selection pressure, plastome structure, nucleotide composition and large- and small-scale rearrangements like segmental deletion or inversions (Wicke et al. 2016; Wicke and Naumann 2018). Our predominantly hemiparasitic sampling of species with apparently different trophic specializations corroborates the generalized mechanistic model of plastome reduction under relaxed selective constraints (Wicke et al. 2016), and, extends this concept with plastome evolution based on trophic transition, though barely observed genomic changes at the semiheterotrophic lifestyle stage.

### Subtle Changes in PS-Associated Genes in Plastomes of Hemiparasites

Most Santalales parasites carry out PS to some extent. Accordingly, we found only subtle changes in PS-associated genes of the analyzed hemiparasitic species, while the holoparasites from Balanophoraceae exhibit the most extensive functional reductions and genomic reconfigurations, including a novel genetic code with a UAG stop codon readthrough (Su et al. 2019). However, changes in selection intensity are seen in many lineages (fig. 3), which relate to both lifestyle transitions and genomic reconfigurations (table 2). The functional and physical gene losses of Santalales are widely convergent with findings from other parasitic angiosperms and in line with predictions according to which *ndh* genes are the earliest-most losses (Wicke et al. 2013, 2016; Barrett et al. 2014, 2018; Feng et al. 2016; Naumann et al. 2016; Braukmann et al. 2017; Barrett and Kennedy 2018; Wicke and Naumann 2018). Despite reports of *ndh* gene losses in some autotrophs (e.g., some gymnosperms and Geraniaceae: Blazier et al. 2011; Chaw et al. 2018; carnivorous Lentibulariaceae and Droseraceae: Martín and Sabater 2010; Wicke et al. 2014; Nevill et al. 2019 or Wicke et al. 2011 for reviews), the number of losses in heterotrophic plants is still exceptionally high. These (independent) loss(es) of *ndh* genes is especially prominent in Santalales, where hemiparasites retain only a few residual segments, but autotrophic species like *E. scandens* harbor intact copies (supplementary fig. S3, Supplementary Material online). Absence of the NADH complex, which mediates electron cycling around photosystem I, causes no severe phenotypic effects unless plants experience light, water, or heat stress (Rumeau et al. 2007). Eco-physiological examination under stress conditions might provide insights whether plants, including hemiparasites, lacking *ndh* genes perform worse than close relatives with an intact NADH complex.

The most prominent PS-associated gene losses of Santalalean hemiparasites are the loss of genes for the PEP (*rpo* genes) and the gene encoding an essential subunit for cytochrome *b* biosynthesis (*ccsA*). The loss of *ccsA* is a novelty and might represent a rather unique, lineage-specific gene loss in photosynthetic Santalales. Further functional examination is needed to test whether cytochrome *b* synthesis is impaired in Santalales, or if the *ccsA* gene might have been functionally transferred to the nuclear genome.

The early loss of PEP on the other hand has been reported earlier in aerial parasites belonging to the genus *Cuscuta* (Funk et al. 2007; McNeal et al. 2007) and in some hemiparasitic Orobanchaceae (Wicke et al. 2013, 2016). PEP transcribes mainly PS genes and is indispensable for proper chloroplast development in autotrophic plants. However, heterotrophic organic carbon that parasitic plants acquire through plant–plant or fungal associations compensates the loss of PEP, which has been experimentally tested by knocking out *rpo* genes in model angiosperms (Pfannschmidt et al. 2015). Studies also have shown that all plastid genes continue to be transcribed in PEP-deficient plants by the second, plastid-specific nuclear-encoded polymerase, albeit at altered transcription levels (Legen et al. 2002). Parasitic *Cuscuta* have also lost PEP promoters but retain transcriptional activity in formerly PEP-transcribed genes (Krause et al. 2003; Berg et al. 2004). Thus, our findings of obligate Santalales hemiparasites not retaining intact *rpo* genes corroborate the hypothesis that *rpo* genes are lost rather early during parasitism-induced reductive plastome evolution because PEP-based transcription might be of less importance for hemiparasites, where a high demand for and rapid turnover of PS genes is reduced (Wicke et al. 2016; Wicke and Naumann 2018).

### Santalales Provide Clues on the Erratic Nature of Plastid HK Gene Losses

Although the pattern of gene losses in Santalales are mostly highly convergent with other parasitic plants in the broader sense (Wicke and Naumann 2018), noting the exceptional changes to the plastid genetic system in Balanophoraceae though (Su et al. 2019), our explicit focus on the transition between various forms of hemiparasitism allowed us to discover a number of previously unseen changes in HK gene contents. We observed the pseudogenization of *clpP* in 13 hemiparasitic species; where present, nonsynonymous and synonymous substitution rates are mildly elevated (fig. 2). The loss of this gene, which codes for a proteolytic core subunit of the plastid Clp protease system, is otherwise lost only in heterotrophs with plastomes of under 50 kb length (Wicke and Naumann 2018), but rare in autotrophs, with the exception of Actinidiaceae (Wang et al. 2016), *Scaevola* (Goodeniaceae, Jansen et al. 2007), and *Passiflora* (Jansen et al. 2007; but see Cauz-Santos et al. 2017). Since the efficient protein quality and removal of damaged or misfolded proteins provided by the ClpP protease is of high importance for functional plastids, especially chloroplasts, we conclude, that *clpP* may have been functionally transferred to the nuclear genome. Alternatively, the plastid copy could have been replaced by a nuclear-encoded isoform (nClpP; Sokolenko et al. 1998; Peltier et al. 2001), which deserves attention in future studies.

Loranthaceae, Opiliaceae, Cervantesiaceae, Santalaceae, Amphorogynaceae, and Viscaceae have no intact gene for the transcription initiation factor I (*infA*), which is essential for the plastid translation machinery. Due to multiple independent functional transfers (Millen et al. 2001), *infA* has become a pseudogene or physically lost from many flowering plant lineages independently, including almost all rosids, some Solanales (Magee et al. 2010; Wicke et al. 2011), and the holoparasite *Conopholis americana* (Orobanchaceae; Wicke et al. 2016). Hence, investigating whether an ancestral gene transfer within the sandalwood order, or evolutionarily earlier, can explain the pseudogenization of plastid *infA* in parasitic Santalales represents an exciting line of follow-up research.

The losses we report here for the ribosomal protein genes *rpl32*, *rpl33*, *rps15*, and *rps16*, which are mainly absent from the plastomes of mistletoe-like parasites, could provide new clues regarding the still enigmatic course of HK gene losses. Although both *rpl33* and *rps15* are known as nonessential for translation, *rps16* and *rpl32* encode indispensable subunits of plastid ribosomes (Zoschke and Bock 2018). However, *rps16* is frequently lost in flowering plants (Wicke et al. 2011), including in many heterotrophs (Wicke and Naumann 2018). Its common pseudogenization and deletion from plastomes can be explained by a substitution of the ribosomal S16 protein through a nuclear gene product that is dual-targeted to both plastids and mitochondria (Ueda et al. 2008). Despite the coexistence of both nuclear and plastid *rps16* over more than a hundred million years, the import of the S16 protein allows the plastid copy to become a pseudogene more often than any other plastid gene, in part due to the loss of splicing activity (Roy et al. 2010).

In contrast, the *rpl32* gene (Fleischmann et al. 2011) has been reported as missing from the plastome only in poplar (Ueda et al. 2007) and a few Ranunculaceae (Park et al. 2015), in both cases as the results of independent functional gene transfers to the nuclear genome. In parasites, its functional or physical loss is seen more frequently, including in stem-feeding *Cuscuta*, especially those with more extensive plastid gene losses, and in fully heterotrophically living plants that exhibit derived stages of plastome degeneration like *Epifagus* (Orobanchaceae; functionally confirmed as a pseudogene by Wolfe et al. 1992) and *Cytinus* (DNA-based annotation as pseudogene by Roquet et al. 2016), *Sciaphila* (Triuridaceae; Lam et al. 2015), *Thismia* (Burmanniaceae; Lim et al. 2016), *Hydnora* (Aristolochiaceae; Naumann et al. 2016), as well as several Orchidaceae (Delannoy et al. 2011; Schelkunov et al. 2015), Ericaceae (Braukmann et al. 2017), and Apodanthaceae (Bellot and Renner 2016). It remains to be investigated whether *rpl32*, and all other losses of HK genes, might be dispensable under heterotrophic conditions or whether these accumulated losses in holoparasitic plants are the result(s) of multiple independent functional replacements. The latter, in combination with the altered selectional pressures under heterotrophic conditions, might underpin the lineage-specific patterns of HK genes losses. However, also existing hypothesis relating to an increased rate of gene transfers (Wicke et al. 2011) should be systematically researched.

In line with recent observations that some tRNAs are apparently lost early during or amidst the specialization on an obligate heterotrophic lifestyle (Wicke and Naumann 2018), we here observed also several tRNA losses in stem-parasitic Santalales. Similar to most Orobanchaceae (Wolfe et al. 1992; Wicke et al. 2013), we found no significant shift regarding the use of specific codons in lineages where certain tRNAs are missing, and, therefore, we may assume that the loss of tRNA isoacceptors is tolerated to some extent under heterotrophic conditions.

### Reductive Plastome Evolution Mirrors the Trophic Transition Series in Santalales

Root-feeding hemiparasites show mildly elevated nucleotide substitution rates and relaxed purifying selection in several photosynthetic genes compared with autotrophs. Stem-parasites exceeded the average substitution rates and numbers of genes experiencing selection relaxation of root-hemiparasitic Santalales, and the mistletoes (*s.l*.) themselves are topped only by the extremely reconfigured, holoparasitic Balanophoraceae (supplementary fig. S10, Supplementary Material online). The selectional shift along the transition to stem-parasitism is especially evident in Viscaceae. These data are in line with a trophic transition series in Santalales, where stem-parasites are known for their relatively high external carbon dependence and carbon acquisition rates compared with the root-feeding parasites (Těšitel et al. 2010; Bell and Adams 2011; Kuijt and Hansen 2015) and the presumed evolution of physiological holoparasitism in some mistletoes (Nickrent and García 2009).

The mushroom look-alike plants belonging to Balanophoraceae are extraordinary regarding their morphology and their molecular evolution. *Balanophora* plastomes are the GC-poorest, functionally active organelle genomes reported to date. Despite the extreme nucleotide compositional bias, protein coding regions continue to evolve under purifying selection and are transcribed due to a novel genetic code (Su et al. 2019). Other highly reduced plastomes like those of *Pilostyles* (Bellot and Renner 2016) and *Hydnora* (Naumann et al. 2016) are characterized by a drastic nucleotide bias with GC contents between 22.7% and 24.2%. It will clearly be of importance for future studies to test whether these plastomes have undergone comparable modification of the genetic apparatus to maintain plastid function. In this sense, Balanophoraceae and Santalales might provide a valuable model system to explore the functional realms of plastids along the transition from an autotrophic to a fully heterotrophic lifestyle.

## Supplementary Material

Supplementary data are available at *Genome Biology and Evolution* online.

## Supplementary Material

evz271_Supplementary_Data

## Notes

**Data deposition:** This project has been deposited at CNSA under the accession CNA0002363-CNA0002393 and GenBank under the accession MN414160-MN414178.
