# Supplementary material for: Comparative Plastome Analysis of Root- and Stem-Feeding Parasites of Santalales Untangle the Footprints of Feeding Mode and Lifestyle Transitions
Source: Genome Biol Evol. 2019 Dec 17;12(1):3663–76. doi: 10.1093/gbe/evz271 (PMC6953812; doi:10.1093/gbe/evz271)
Supplement: evz271_Supplementary_Data [file evz271_supplementary_data.zip › Legends of supplementary figures and tables.docx]

**Supporting information**

Additional supporting information may be found in the online version of this article.

**Fig. S1** Paired-end (PE) reads mapping to the assembly of *Taxillus chinensis*, the connections between LSC, IRb, SSC and IRa were marked seperetely. Red curves indicated insert size ranged 100-400bp, blue curves indicated insert size ranged 400-1,000bp, deeppink curves indicated insert size ranged 40kbp-100kbp, navy curves indicated insert size ranged 100kbp-1Mbp.

**Fig. S2** The pairwise alignment between our assembly and published assembly based on the same species. One of the IR region of our assembly was blocked before alignment. The red color indicated coserved regions between them, while the blue color indicated reverse-compliment alignment.

**Fig. S3** Locally co-linear blocks (LCBs) identified by MULTIZ. Plastid genomes of all plants were depicted as linear maps, with gene boxes on both left and right. Genes were colored according to their functional classes at the bottom. Locally colinear blocks (LCBs) derived from whole-plastome alignments were illustrated as large colored blocks, and each block is represented with a different color. Changes in the orientation of LCBs relative to other species mark inversions. Similarity plots within the LCBs indicated the degree of sequence conservation between species, whereas long bars represent high sequence similarity. (a) LCBs in nonparasitic and hemiparasitic plants. (b) LCBs in holoparasitic plants analyzed in this paper.

**Fig. S4** Linear map and GC content of the plastid chromosome of Balanophoraceae. Genes drawn on the upper side are transcribed from left to right, and on the lower side, from right to left. The positions of introns are shown with inverse ‘V’. The histogram shows GC content that is greater than (upper the line) or less than (below the line) the average GC content of each plastome. The gene contents in *Rhopalocnemis phalloides*, *Balanophora reflexa*, and *Balanophora laxiflora* were drawn based on NCBI No. MK036331, KX784266, and KX784265.

**Fig. S5** Gene arrangement comparison between studied species. For each plastome genome, the protein-coding genes were displayed by locations, whose corresponding fill color by four regions: LSC (lime), IRb (teal), SSC (cyan) and IRa (teal). With red and blue framed boxes indicated the direction of transcription (plus and minus, respectively). ‘x’ and ‘+’ within the boxes indicated the loss and gain genes compared with the NP *Vitis rotundifolia*.

**Fig. S6** GC Contents and variation at different codon positions of protein-coding genes in plastomes of Santalales and outgroups. Upper, GC content at different codon positions of intact plastid protein-coding genes of the nonparasitic and parasitic plants. Below, Variations among GC content at different codon positions in coding regions of parasites and nonparasites assessed as the difference (D value) to a reference genome (*Tetrastigma hemsleyanum*, KT033563). A line inside each box designates the median; the whisker ends are at the 5th and 95th percentiles.

**Fig. S7** Proportions of different repeat types in Santalales plastomes with REPuter.

**Fig. S8** Distribution of different repeat length in Santalales plastomes.

**Fig. S9** Self-self dot plots of plastomes in Santalales illustrated the number and distribution of small and large repeats. Direct repeats are illustrated as red dots or lines whereas inverted repeats are shown as blue dots or lines.

**Fig. S10** Selectional changes per branch across all universal protein genes including Balanophoraceae. Only 8 genes shared in all species were used. The genes are color-coded according to the selection strength parameter k, inferred under the general descriptive RELAX model. Low k (blue) indicates a relaxation of purifying selection, whereas high k (red) suggests the selection intensification. The phylogenetic tree and scales come from Fig. 1.

**Table S1** The species, sources, lifestyles and feeding modes of Santalales analyzed in this study.

**Table S2** The custome plastome dataset used for annotation or codon usage comparison.

**Table S3** Overview of physical properties in plastomes of Santalales.

**Table S4** Overview of coding and noncoding regions in plastomes of Santalales.

**Table S5** Number of different repeat types in plastomes of Santalales with REPuter.

**Dataset S1** Gene information in plastomes of Santalales.

**Dataset S2** Sginificant changes of nucleotide compositions of genes among different lifestyles and feeding modes based on phylogenetic relationship

**Dataset S3** Lifestyle and feeding modes effect on selection in functional gene complexes in Santalales plastomes (Balanophoraceae excluded).

**Dataset S4** Lifestyle and feeding modes effect on selection in functional gene complexes in Santalales plastomes (Balanophoraceae included).
