# Supplementary figures and images for: Comparative Plastome Analysis of Root- and Stem-Feeding Parasites of Santalales Untangle the Footprints of Feeding Mode and Lifestyle Transitions
Source: Genome Biol Evol. 2019 Dec 17;12(1):3663–76. doi: 10.1093/gbe/evz271 (PMC6953812; doi:10.1093/gbe/evz271)

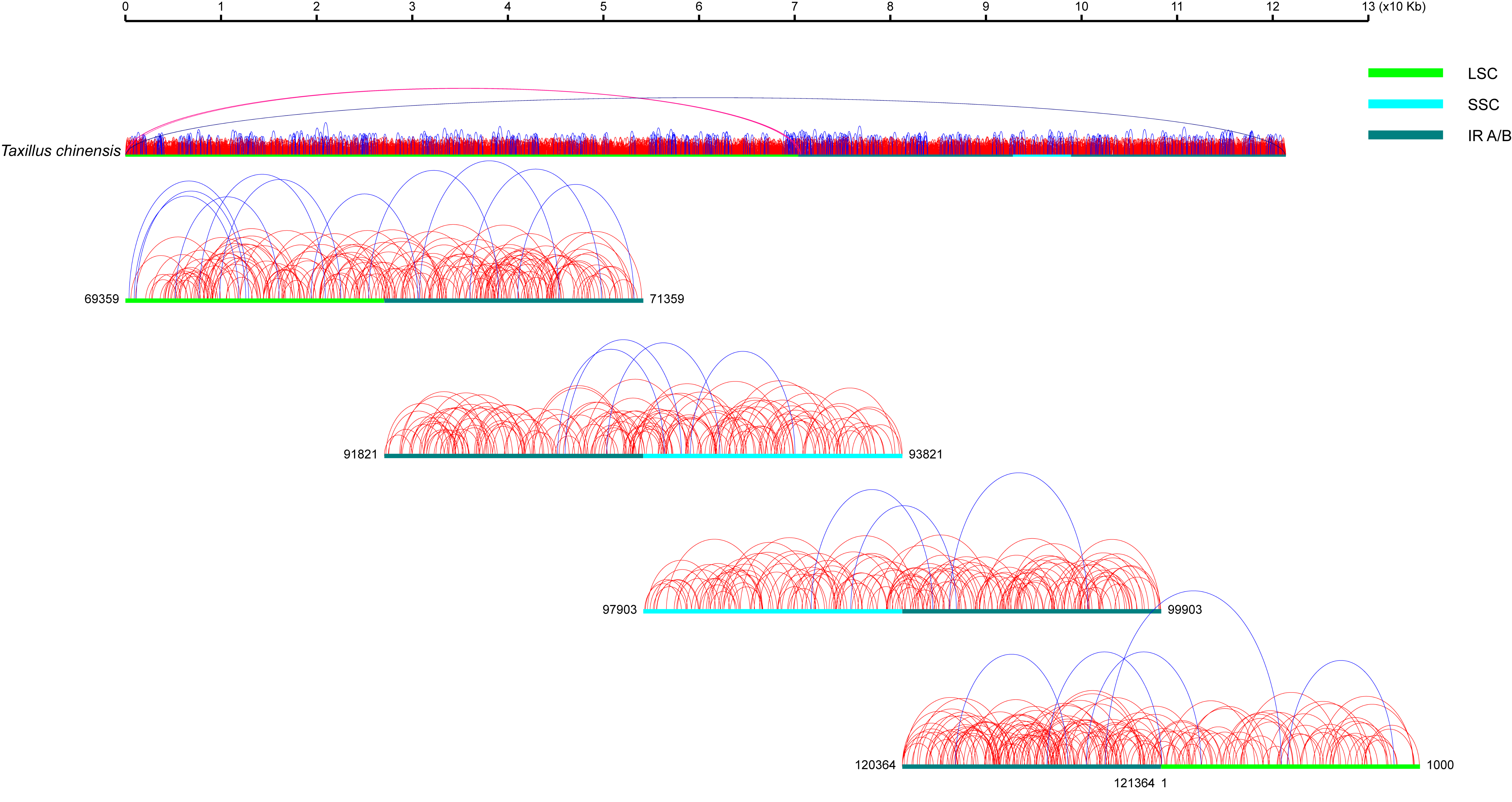

Supplement: evz271_Supplementary_Data [file evz271_supplementary_data.zip › Fig. S1.tiff]

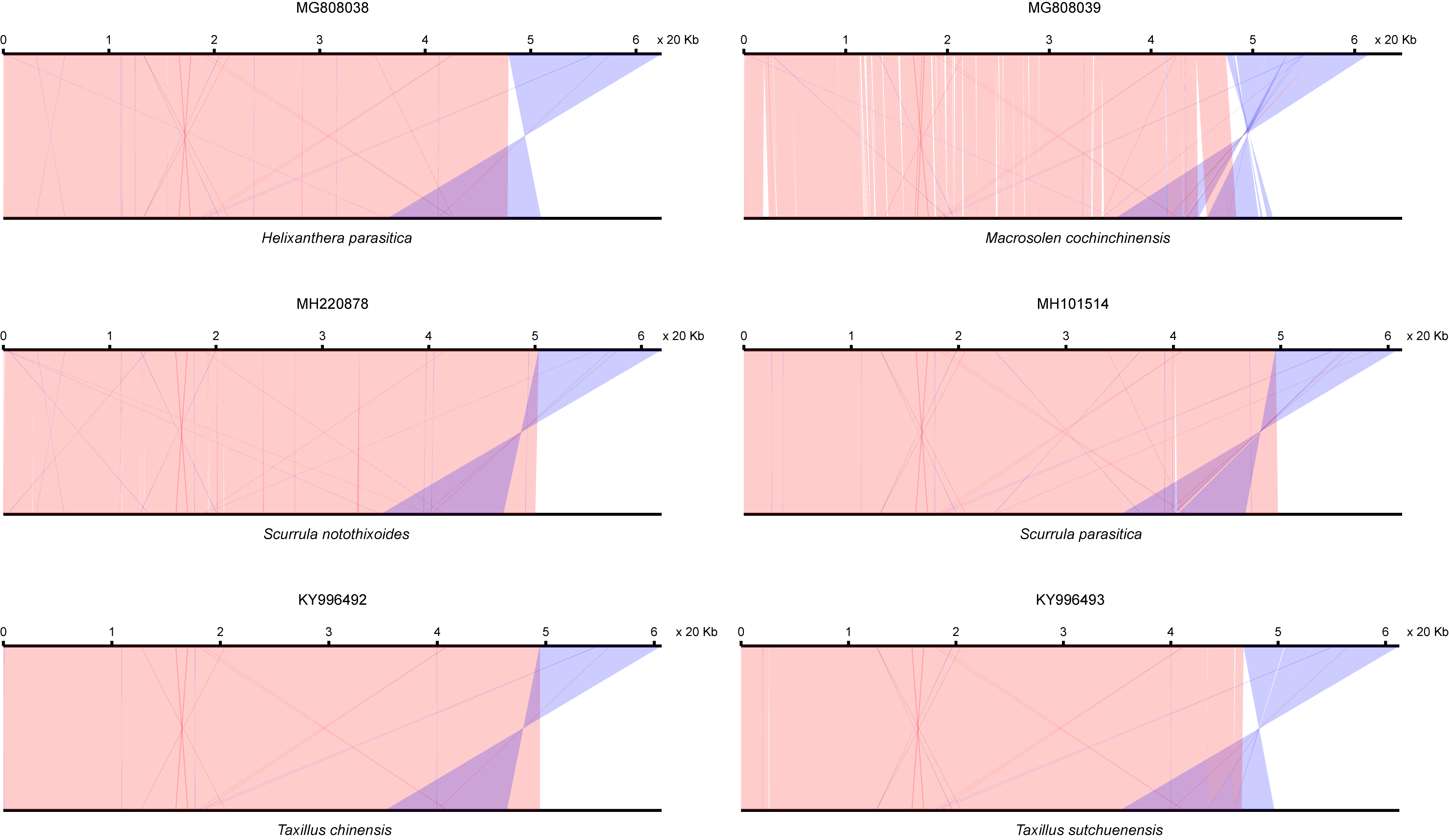

Supplement: evz271_Supplementary_Data [file evz271_supplementary_data.zip › Fig. S2.tif]

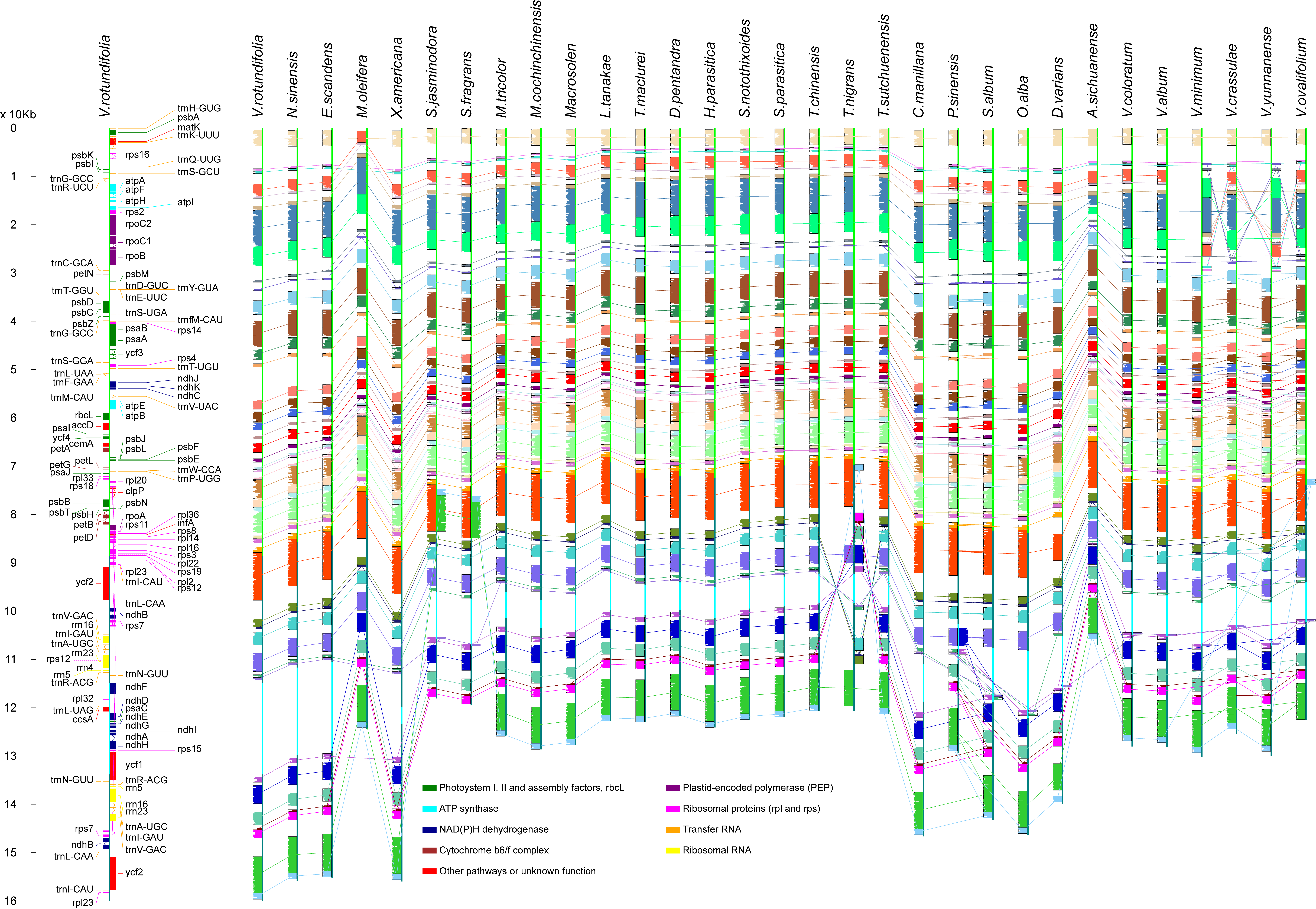

Supplement: evz271_Supplementary_Data [file evz271_supplementary_data.zip › Fig. S3(a).tif]

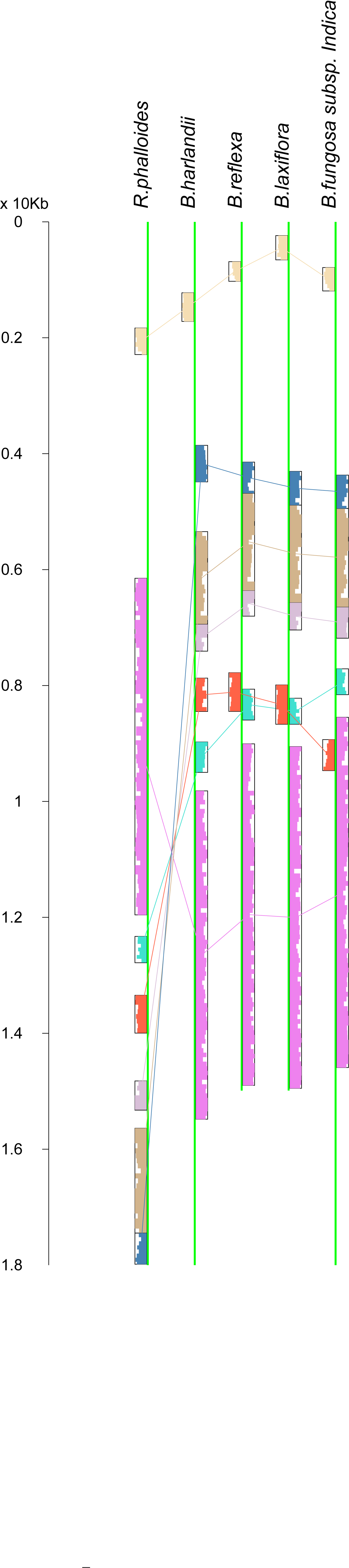

Supplement: evz271_Supplementary_Data [file evz271_supplementary_data.zip › Fig. S3(b).tif]

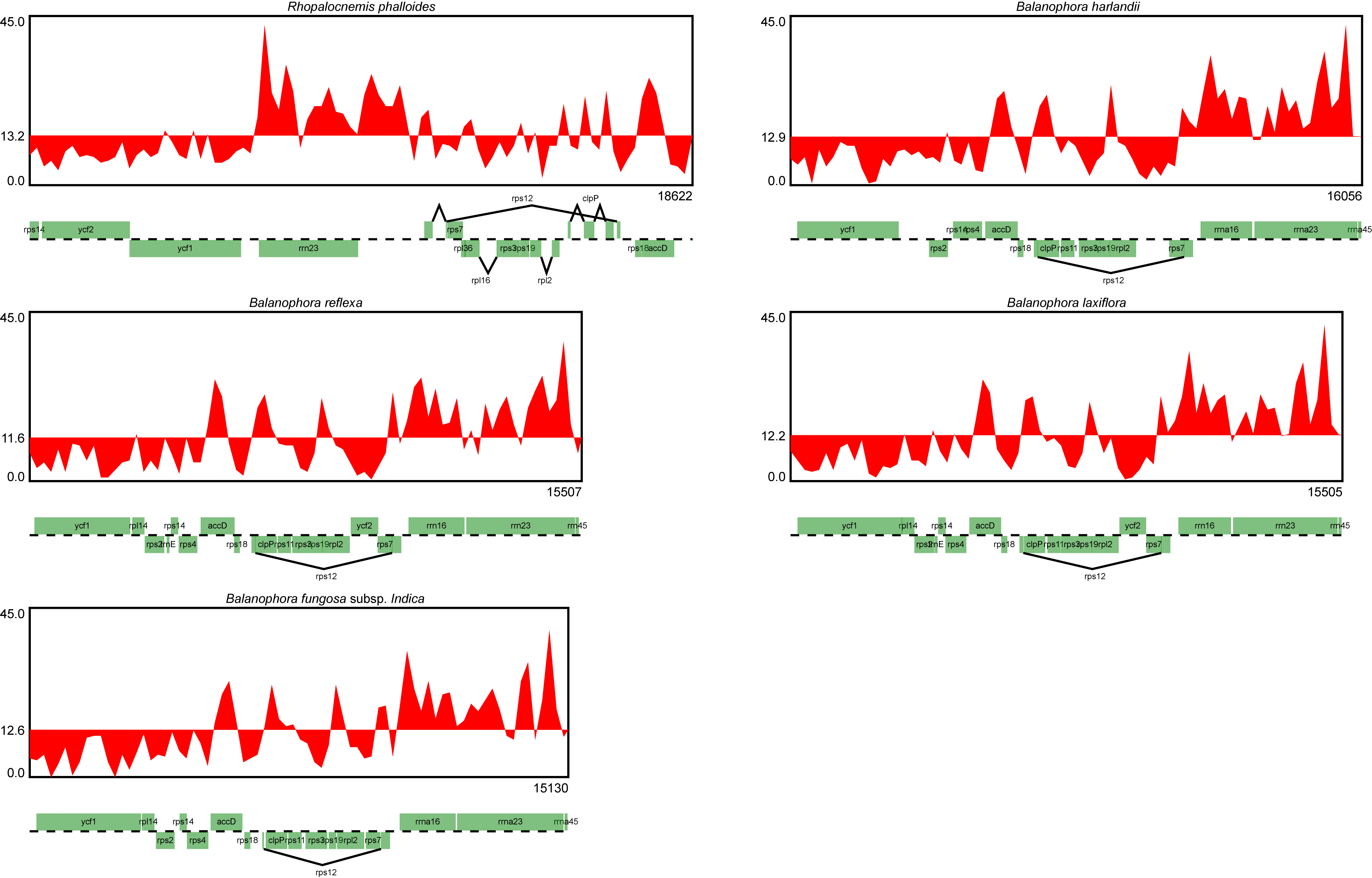

Supplement: evz271_Supplementary_Data [file evz271_supplementary_data.zip › Fig. S4.tif]

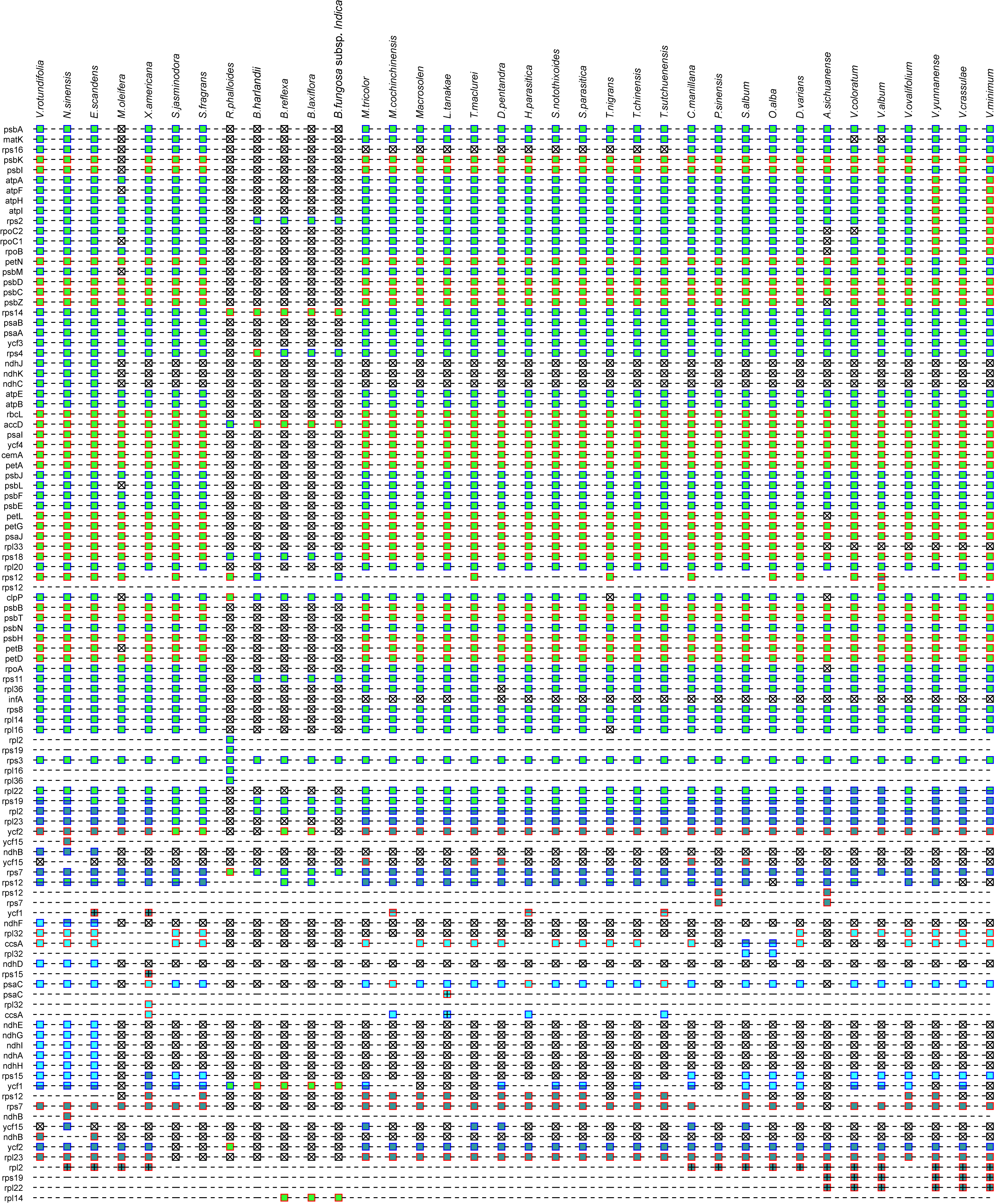

Supplement: evz271_Supplementary_Data [file evz271_supplementary_data.zip › Fig. S5.tif]

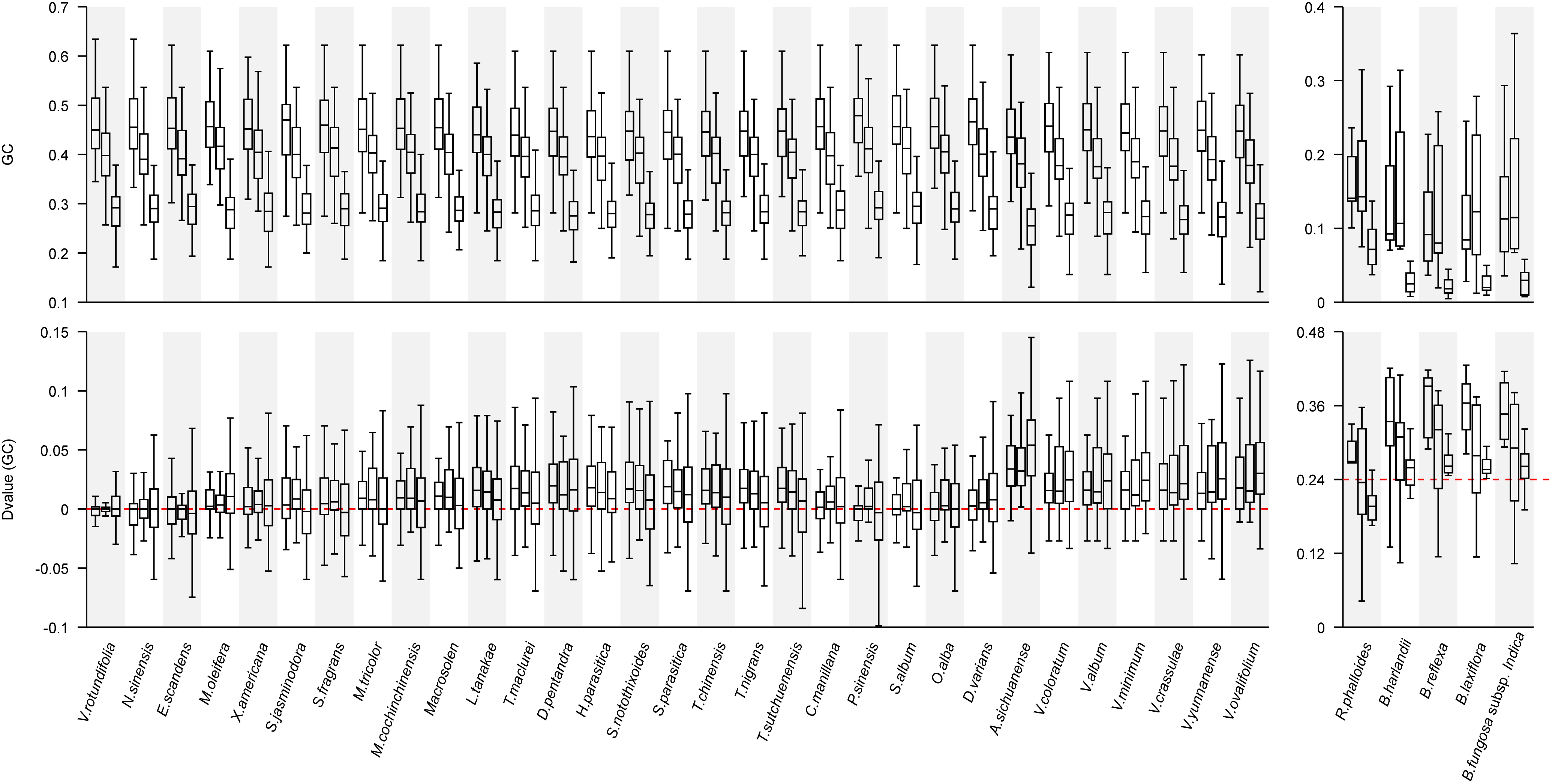

Supplement: evz271_Supplementary_Data [file evz271_supplementary_data.zip › Fig. S6.tif]

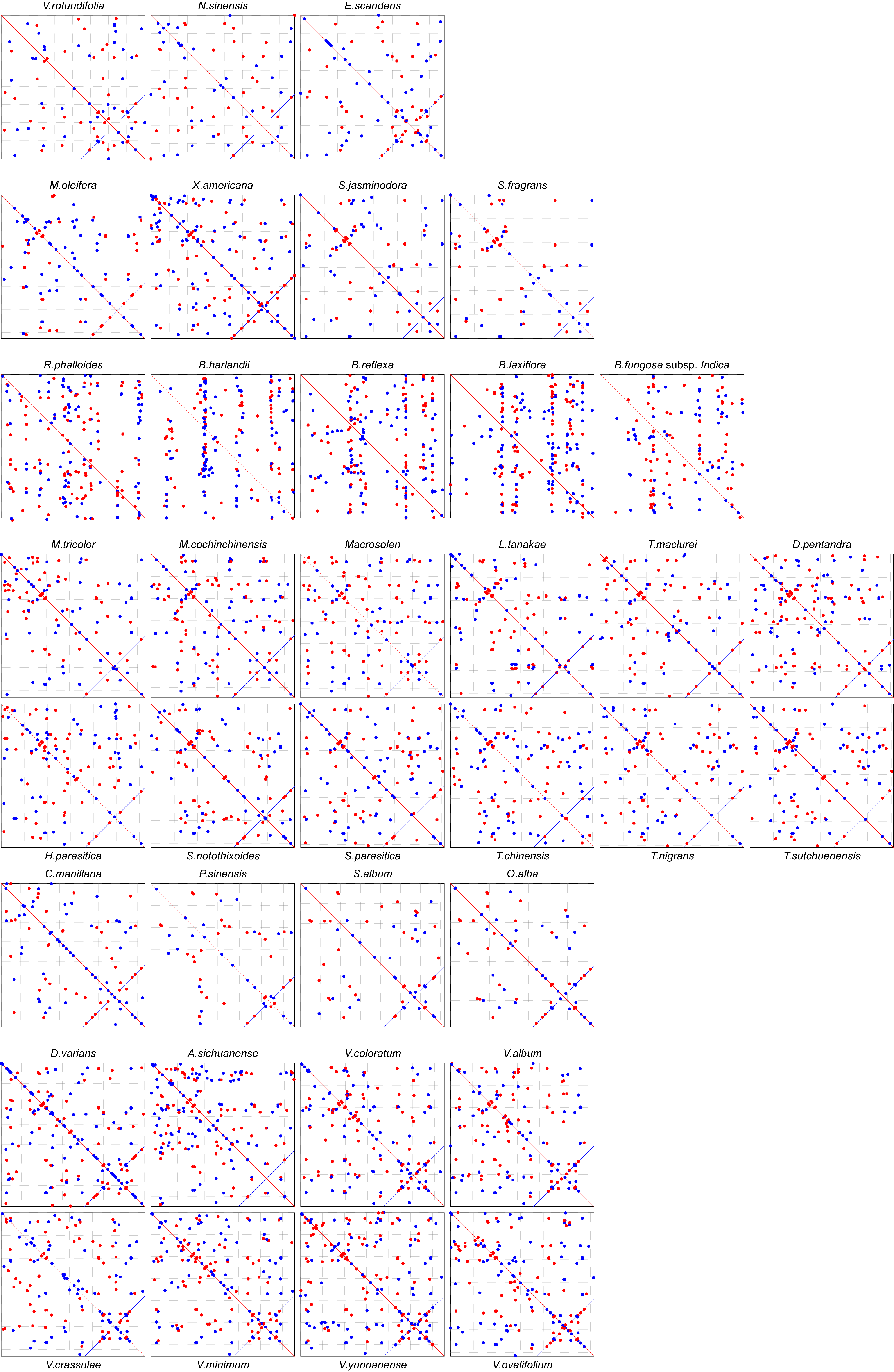

Supplement: evz271_Supplementary_Data [file evz271_supplementary_data.zip › Fig. S9.tif]
